# Ipsilateral Simultaneous Low-Energy Multiple Upper Limb Fractures in an Elderly Patient: Case Report and Review of the Literature

**DOI:** 10.1155/2022/3571724

**Published:** 2022-05-18

**Authors:** Dany Aouad, Rabih Kortbawi, Mohammad Daher, Alexandre Nehme, Ramzi Moucharafieh, Mohammad Badra

**Affiliations:** ^1^Department of Orthopedic Surgery and Traumatology, Saint Georges University Medical Center, Balamand University, P.O. Box 166378, Achrafieh, Beirut 1100 2807, Lebanon; ^2^Faculty of Medicine, University Saint Joseph, P.O. Box 17-5208, Mar Mikhael, Beirut, Lebanon; ^3^Department of Orthopedic Surgery and Traumatology, Clemenceau Medical Center Affiliated with Johns Hopkins International, Clemenceau Beirut, Lebanon

## Abstract

Traumatic injuries of the upper limb can result in variant fracture combination. This article discusses a rare injury combination including ipsilateral proximal and distal humerus fractures alongside a distal radius fracture. The mechanism of the fall is unknown, but the patient being old with such a complex injury, one can only assume that osteoporosis played a major role. Open reduction and internal fixation was opted for the distal humerus and radius fractures, and percutaneous pinning was done for the proximal humerus fracture. Surgery is an option to each one of these injuries with different techniques available for managing such an association, with emphasis made on osteoporosis workup to help prevent such complex injuries.

## 1. Introduction

Traumatic injuries of the upper limb can result in an association of multiple injuries, and this combination is usually dependent on the mechanism of injury as well as other factors. The most common orthopedic fracture concerns the distal radius, and they usually result from a fall on outstretched hand [1]. Proximal humerus fractures are the third most common nonvertebral fracture pattern seen in the elderly, and they too usually result from the same mechanism mentioned above [2, 3]. Supracondylar humeral fractures account for around 0.5 to 0.7% of all orthopedic fractures, and they usually result from low-energy falls in elderly and high-energy impact in young individuals [4–7]. Different combinations of fractures involved in the ipsilateral upper extremity were described in the literature. This article reports a case about a combination of a proximal and distal humerus with a distal radius fracture which to the best of our knowledge has not been reported in the literature yet.

## 2. Case Report

This is a case of an 80-year-old female patient who presented to the emergency room (ER) after slipping and falling from height in the shower. Her past medical history consists mainly of medically controlled hypertension and osteoporosis. The patient presented with a head laceration that needed suturing and with severe right upper extremity pain. She does not recall the mechanism of the fall. No loss of consciousness was reported. On physical examination, the shoulder was adducted and internally rotated, with an apparent deformity and swelling of the elbow and wrist joints. Tenderness with decreased range of motion in all three joints was noted. Neurovascular exam was intact.

**Figure 1:**
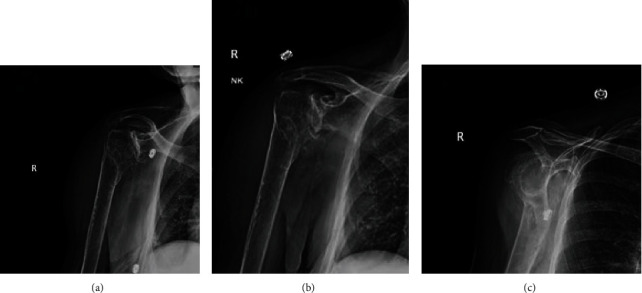
(a–c) Plain radiographs of the right shoulder showing a displaced proximal humerus fracture involving the surgical neck and greater tuberosity.

**Figure 2:**
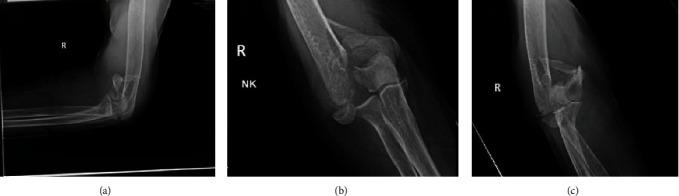
Plain radiograph of the right elbow with (a) the lateral view showing a comminuted distal humeral fracture, (b) AP view showing a bicondylar distal humeral displaced fracture, and (c) showing the right elbow with a two column distal humerus fractures classified as lateral lambda as per the Jupiter classification.

**Figure 3:**
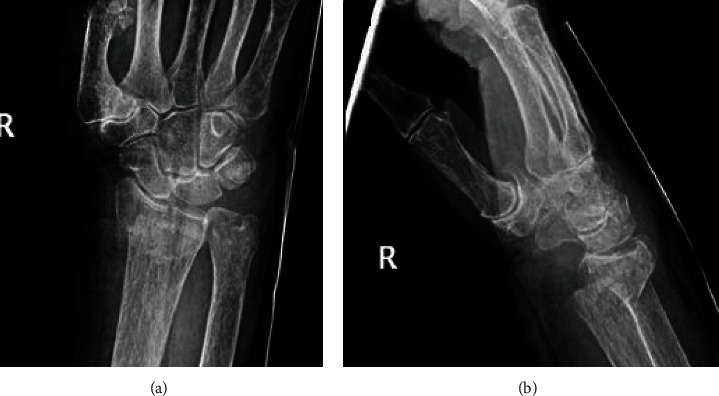
Plain radiographs of the right wrist in the (a) AP and (b) lateral views showing a dorsally displaced distal radius fracture with intra-articular extension.

Brain CT scan was done showing no intracerebral bleeding or acute abnormalities. Plain radiographs of the right shoulder (Figure 1), elbow (Figure 2), and wrist (Figure 3) showed a displaced proximal humerus fracture, a distal humerus fracture, and a dorsally displaced distal radius fracture, respectively.

In the ER, an above-elbow posterior splint was applied that extended from the volar metacarpophalangeal joint up to the tip of the shoulder with the wrist in neutral position, the elbow flexed to 90 degrees, and the shoulder adducted and internally rotated, placed in an arm sling.

**Figure 4:**
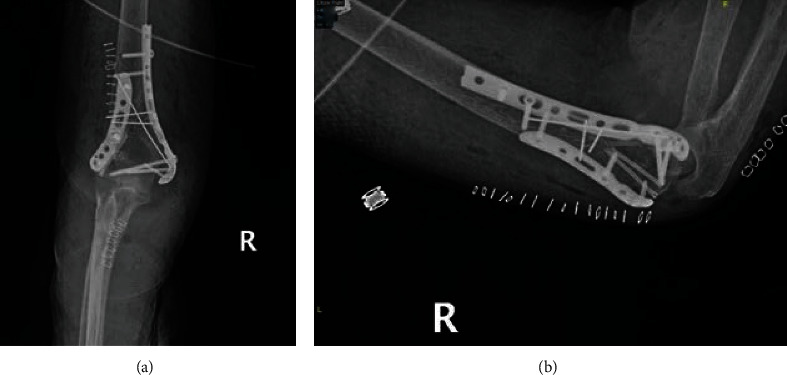
Postoperative plain radiographs of the right elbow showing on the (a) AP and (b) lateral views reduction of the medial and lateral epicondyles using double plating with ten screws.

Decision was taken to opt for surgery through open reduction and internal fixation. With the patient placed in a prone position, the elbow was approached posteriorly. After adequate exposure of the fracture site, reduction of the medial and lateral epicondyles was performed with temporary fixation using k-wires. Double plating was then used with ten screws for definite fixation (Figure 4).

**Figure 5:**
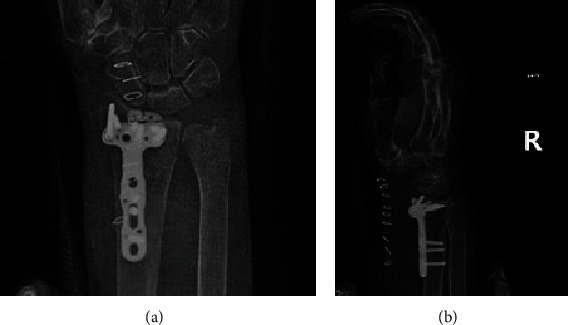
Postoperative plain radiographs of the right wrist showing on the (a) AP and (b) lateral views reduction of the distal radius fracture using a distal volar plate and nine screws.

The patient's position was then changed to supine, where a volar approach was performed to the wrist with an incision over the flexor carpi radialis tendon. The fracture was reduced and fixed using a distal volar plate and nine screws (Figure 5).

**Figure 6:**
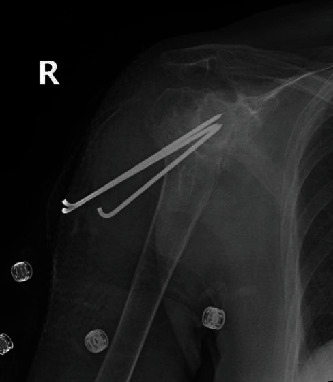
Postoperative plain radiographs of the right shoulder showing percutaneous pinning of the proximal humerus fracture using three k-wires.

The patient was then placed in the beach chair position. Closed reduction and percutaneous pinning of the proximal humerus fracture was performed using three k-wires (Figure 6). Following surgery, the arm was placed in an arm sling.

Upon follow-up at one, three, and six months, respectively, the patient improved progressively with return to preoperative daily activities after multiple sessions of physical therapy and does not report residual pain, with no alteration of shoulder, elbow, and wrist range of motion.

## 3. Discussion

Since neither the fall was witnessed nor the patient could recall the exact mechanism of injury, one could only hypothesize that the fall from height was probably on an outstretched arm, the elbow flexed to 90 degrees, the forearm pronated, and the wrist hyperextended. Having a combination of two of these three fractures present in the same individual is rare. Thus, having a patient with a proximal humerus, a supracondylar elbow, and a distal radius fracture in the ipsilateral upper extremity is extremely rare and is probably related to her preexisting osteoporosis. It is believed that having all three fractures in the same upper limb with at least one of the fractures being unstable reduces the chances of conservative treatment and requires internal fixation followed by early rehabilitation program. The literature still lacks a consensus on the order of treatment to follow when dealing with multiple fractures in the upper limb. In this case, the first injury to be treated was the one found to be the most severely deformed, the distal humerus fracture. Once the elbow was stabilized, reduction and fixation of the distal radius and proximal humerus was practically more suitable.

The distal humerus can be simplified into three columns, transverse intercondylar, medial, and lateral [8]. The integrity of this part of the humerus depends on the integrity of the triangle formed by the latter columns [8]. The recommended treatment of such fractures is double plating although Kumar et al. showed good results using a single plate [9]. The position of the plates can be either parallel or orthogonal. Studies did not show significant differences between these positions [8]. Parallel plating is recommended in fractures with extensive intra-articular damage which was the case in the above described patient [8]. The use of as many screws as possible is also recommended for better biomechanical stability [8]. In the distal radius fracture plate, nine screws were used, six of which were in the distal volar plate. Mansuripur et al. investigated the effect of the number of screws used on the outcome [10]. There is not any significant differences when seven screws were compared to four, but the higher the number of screws there is the higher risk of extensor irritation and intra-articular placement [10]. For osteoporotic intra-articular distal radius fracture managed with a volar plate and submaximal distal screws, Mansuripur et al. showed that the construct is stable enough for early postoperative rehabilitation [10]. In the abovementioned case, early active assisted wrist range of motion exercises were started at two weeks postoperatively. The treatment of proximal humerus fracture in osteoporotic patients can be either conservative or operative with no difference in outcome [11]. If the injury is not open or associated to neurovascular damage, the indications of operative management are controversial [11]. In case operative management was chosen, it is recommended to start passive motion of the shoulder to aid in the healing process [11]. Some studies have shown that the use of screws in osteoporotic patients is not that good of an option due to the reduced holding strength of the screws [12]. The plan in the described case was to temporarily fixate this fracture, while the other injuries were managed and given time to heal before moving on to reverse total shoulder arthroplasty (RTSA). RTSA has become the best treatment for unreconstructible fractures in the proximal humerus in elderly patients with associated degenerative rotator cuff arthropathy [11].

## 4. Conclusion

This article reports a combination of ipsilateral proximal and distal humerus fracture with a distal radius fracture. For bicolumnar fractures in the distal humerus, double plating especially parallel is the gold standard when there is extensive intra-articular damage. Operative management with stable mechanical fixation in multiple upper limb fractures is required in the aim of early range of motion exercises, both to regain function and to prevent immobilization for longer periods of time, which may cause stiffness and impairment. Further treatment algorithms should be introduced concerning the order of treatment of upper limb concomitant multiple fractures, especially in elderly osteoporotic patients. Emphasis should be made on the importance of osteoporosis screening, diagnosis, and treatment, mainly in postmenopausal women, aiming to prevent such multiple extensive injuries.

## Data Availability

The data used to support the findings of this study are available from the corresponding author upon request.
